# Femoral periprosthetic fracture treatment using the Ortho-Bridge System: a biomechanical study

**DOI:** 10.1186/s13018-022-03154-w

**Published:** 2022-06-03

**Authors:** Yuntao Long, Yubin Qi, Guilai Zuo, Qingjie Zhang, Zhenlin Liu, Wen Wang

**Affiliations:** 1 Shandong First Medical University & Shandong Academy Medical Sciences, Jinan, 250117 Shandong China; 2 Department of Orthopaedics, The First Affiliated Hospital of Shandong First Medical University & Shandong Provincial Qianfoshan Hospital, Jinan, 250014 Shandong China; 3 Newton Laboratories, Tianjin Weiman Biomaterials Co., Ltd, Tianjin, 301600 China

**Keywords:** Biomechanical study, Ortho-Bridge System, Locking Compression Plate, Locking Attachment Plate, Vancouver B1 periprosthetic fracture

## Abstract

**Background:**

We undertook a comparative biomechanical study of type B1 fractures around femoral prostheses following cemented hip arthroplasty using the Ortho-Bridge System (OBS) and a locking compression plate/locking attachment plate structure (LCP + LAP). We aimed to investigate the biomechanical characteristics and advantages of the OBS compared with LCP + LAP when treating this fracture type.

**Methods:**

An OBS fixation model was designed based on OBS and LCP + LAP fixation characteristics. The LCP + LAP combination (Group A) and three different OBS combinations (Groups B, C, and D) were used to fix a B1 fracture model with a femoral periprosthetic fracture. Axial compression and torsion experiments were then performed using simple and comminuted fracture models. The axial compression failure experiment was carried out, and the model stiffness during axial compression, torsion angle in torsion test, and vertical load in the final failure test were collected.

**Results:**

When simulating simple oblique fractures, no significant difference was found among the four groups in terms of stiffness in the axial compression experiment (*P* = 0.257). The torsion angle of the LCP + LAP system was significantly higher compared with the OBS system (*P* < 0.05). When simulating a comminuted fracture, the experimental data for axial compression showed that the rigidity measurements of the three combinations of the OBS system were higher compared with the LCP + LAP system (*P* = 0.000) and that the torsion angles of three combinations of the OBS system were smaller compared with the LCP + LAP system (*P* < 0.05). In the axial compression failure test, the fixed failure mode of the LCP + LAP system was the destruction of the contact cortex at the fracture site, whereas the failure modes in the three OBS combinations involved fracture around the screws above the osteotomy and destruction of the contact cortex at the fracture site.

**Conclusions:**

The findings revealed that the OBS produced superior biomechanical outcomes compared with LCP + LAP, especially for the bridging two-rod dual cortex. According to the performance observed after model axial compression destruction, the OBS was fixed and provided greater stress dispersion, which might make it more suitable for facilitating early functional movement and avoiding the failure of internal fixation.

## Background

In 1954, Holovitz and LeNobel first described periprosthetic fractures (PPFx) as those that are above, below, or around implant prostheses [1]. The number of hip replacement surgeries reported by the National Joint Registry has increased annually, and 91,833 such operations were conducted in 2015 [2]. The rise in PPFx is directly related to the increasing frequency of primary joint replacement operations every year [3]. With an increase in the number of hip replacements, the number of PPFx during and after surgery is expected to increase [4–7]. According to related reports, the incidence of PPFx is 11% after the first total hip replacement and 7% after a semi-joint replacement [8, 9].

The Vancouver classification proposed by Duncan [10] is the most commonly used classification for femoral PPFx and includes the anatomical site of the fracture, prosthesis stability, and bone stock quality, which is helpful information when determining a final fracture treatment plan [11]. When femoral prostheses are well fixed (type A, type B1, and type C), PPFx can be treated either non-surgically or through an open reduction and internal fixation (ORIF) procedure, whereas types B2 and B3 require prosthesis revision [4, 12, 13]. The most common treatment for type B1 PPFx using the Vancouver classification has been reported to be an ORIF procedure using locking plates, steel cables, or allogeneic cortical supports [13].

**Fig. 1:**
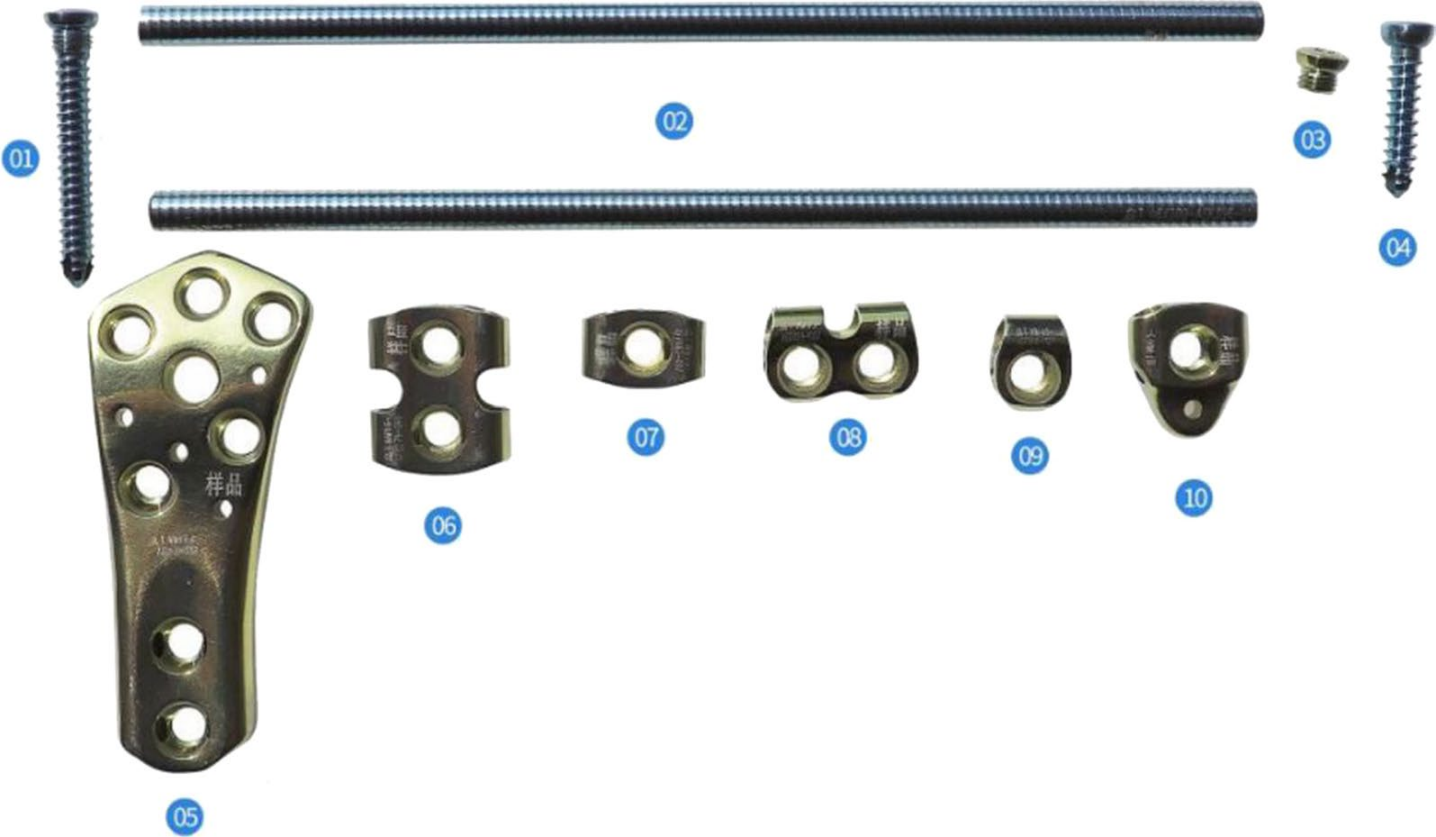
Basic unit components of the OBS. (**01**) Locking screws, (**02**) connecting rod, (**03**) locking nut, (**04**) ordinary screws, (**05**) distal shaped piece of the femur, (**06**) double-rod double-hole fixing block, (**07**) double-rod single-hole fixing block, (**08**) single-rod double-hole fixing block, (**09**) single-rod and single-hole fixing block, and (**10**) end block fixing block

**Fig. 2:**
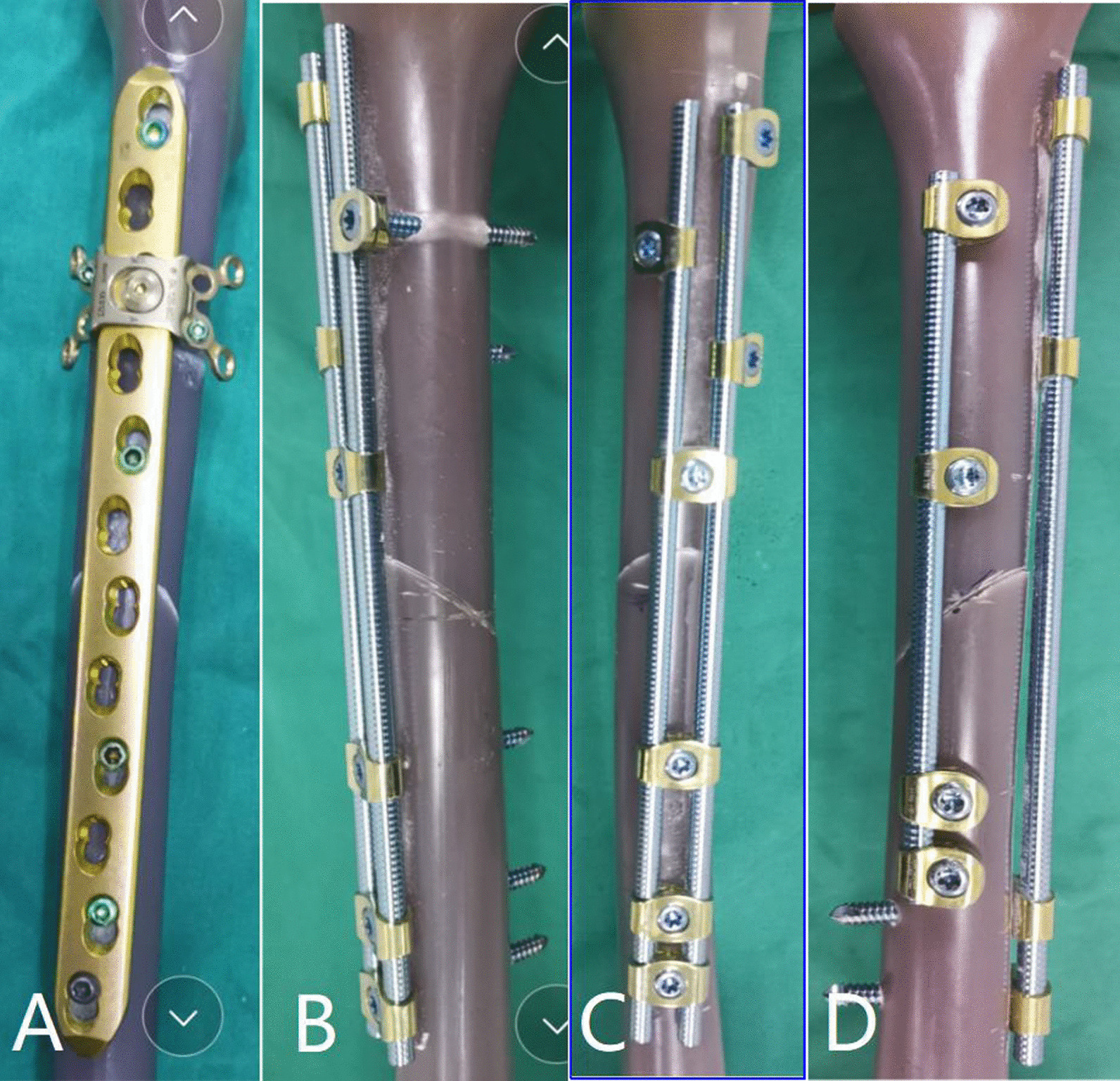
Steel plate and bridge fixing model structure. Group **A**, LCP + LAP fixing group; Group **B**, bridging double-rod double-cortex fixation group; Group **C**, bridging double-rod single-cortex fixation group; Group **D**, bridging single-rod cross-fixation group

Although the main treatment for Vancouver B1 fractures is open reduction and internal fixation, no consensus has been reached concerning the optimal method of reduction and fixation [6, 14–16]. The Ortho-Bridge System (OBS) is a new internal fixation system [17, 18] developed by Tianjin Weiman Biomaterials Co., Ltd. It is made of a titanium alloy and consists of connecting rods, locking screws, locking nuts, fixing blocks, and common screws (Fig. 1). As the basic units of the OBS, nails, rods, and blocks can be individually combined, configured, and fixed using either single, double, or multiple rods. Moreover, the OBS can be locked and unlocked. The individualised combination of nails and rods makes it possible to have diverse fixed positions. At the same time, it allows three-dimensional (3D) fixation with multi-bar and steering nails, improves the pull-out strength, and creates a larger personalised application space for the treatment of particularly complex fractures. Biomechanical and clinical analysis studies have shown that internal fixation using the OBS was effective in treating long bone fractures [17, 19]. Moreover, the OBS has been applied in the fixation of the upper and lower limbs and pelvic fractures. Some orthopaedic surgeons in China have attempted to use the OBS to treat femoral PPFx. Although good results have been achieved, knowledge of this application of the OBS is greatly limited due to a scarcity of published data. Therefore, in this study, we refer to various previously designed PPFx internal fixation concepts, including titanium cable, steel-wire cerclage and locking plate, among others [20]. The fixed-angle internal fixation or construct has been shown to be consistently stronger against pull-out forces and deformation [21] because it uses a bicortical screw channel in the proximal femur for fixed-angle fixation. Based on the advantages of the OBS, we designed a PPFx model of the OBS and conducted biomechanical analysis and comparison experiments with the model, which was made using the LCP + LAP fixation system that is in common use worldwide (Fig. 2).

This study aimed to investigate the biomechanical characteristics and advantages of the OBS compared with the LCP + LAP system in a Vancouver B1 femoral periprosthetic fracture model and to find the theoretical basis for using the OBS to treat periprosthetic fracture so as to assist clinical research.

## Methods

### Experimental materials

The following materials were used in this study: 24 artificial standard femora (third-generation compound femora, medium 3304; Sawbones [22, 23], a department of the Pacific Research Laboratory in Vashon, Washington); a 12-hole Synthes plate and a corresponding attached plate (DePuy Synthes, Solothurn, Switzerland); and the ortho-bridge system, comprised a 6-mm-diameter connecting rod, a fixing block, and a fixing screw (Tianjin Weiman Biomaterials Co., Ltd., Tianjin, China).

### Experimental grouping

This experiment comprised the four following groups: (1) Group A: fixed LCP + LAP; (2) Group B: bridging double-rod and double-cortex fixation; (3) Group C: bridging double-rod and single-cortex fixation; and (4) Group D: bridging single-rod cross-fixation. Six experimental models were constructed for each group (Fig. 2). The number of experimental models was determined in accordance with similar experiments undertaken internationally [24] and also met requirements for statistical analysis.

### Model construction

This experiment was completed in the Newton Laboratory of Tianjin Weiman Biomaterials Co., Ltd. Standard femoral neck osteotomy was performed on 24 Sawbones third-generation compound femora using a swing saw positioned 10 mm from the proximal end of the trochanter. The opening was chiseled, then the medullary cavity was widened with a medullary cavity file, and an appropriate medullary cavity expander was selected to enlarge the medullary cavity. A cement restrictor was placed at the distal end of the medullary cavity, which was manually filled with an appropriate amount of bone cement, and the prepared femoral stem prosthesis was positioned. The femoral stem prosthesis model was prepared for internal fixation using the 12-hole Synthes plate and the corresponding attached plate and OBS system. The length of the OBS connecting rod was consistent with a standard 12-hole locking plate, which was placed at the planned position under the femoral trochanter. In the bridging double-cortex fixation group, the bicortical screw channel at the upper end of the fracture was located under fluoroscopic observation (Fig. 2). The specific fixing requirements of each group were as follows:

1. Group A: LCP + LAP fixation group

A 12-hole Synthes locking compression plate and an attachment plate were used. Proximal fixation was achieved with two double-cortex locking screws and two single-cortex locking screws. Distal fixation was achieved with three double-cortex locking screws. The screws were 5 mm in diameter.

(2) Group B: bridging double-rod and double-cortex fixation

Two connecting rods (length, 22 cm; diameter, 6 mm) were used. Proximal fixation was achieved with three single-rod single-hole fixation blocks, a double-rod single-hole fixation block, three double-cortex locking screws, and a single-cortex locking screw. Distal fixation was achieved with three double-rod single-hole fixation blocks and three double-cortex locking screws.

(3) Group C: bridging double-rod single cortex fixation

Two connecting rods (length, 22 cm; diameter, 6 mm) were used. Proximal fixation was achieved with three single-rod single-hole fixation blocks, a double-rod single-hole fixation block, and four single-cortex locking screws. Distal fixation was achieved with three double-rod single-hole fixation blocks and three double-cortex locking screws.

(4) Group D: bridging single-rod cross-fixation

**Fig. 3:**
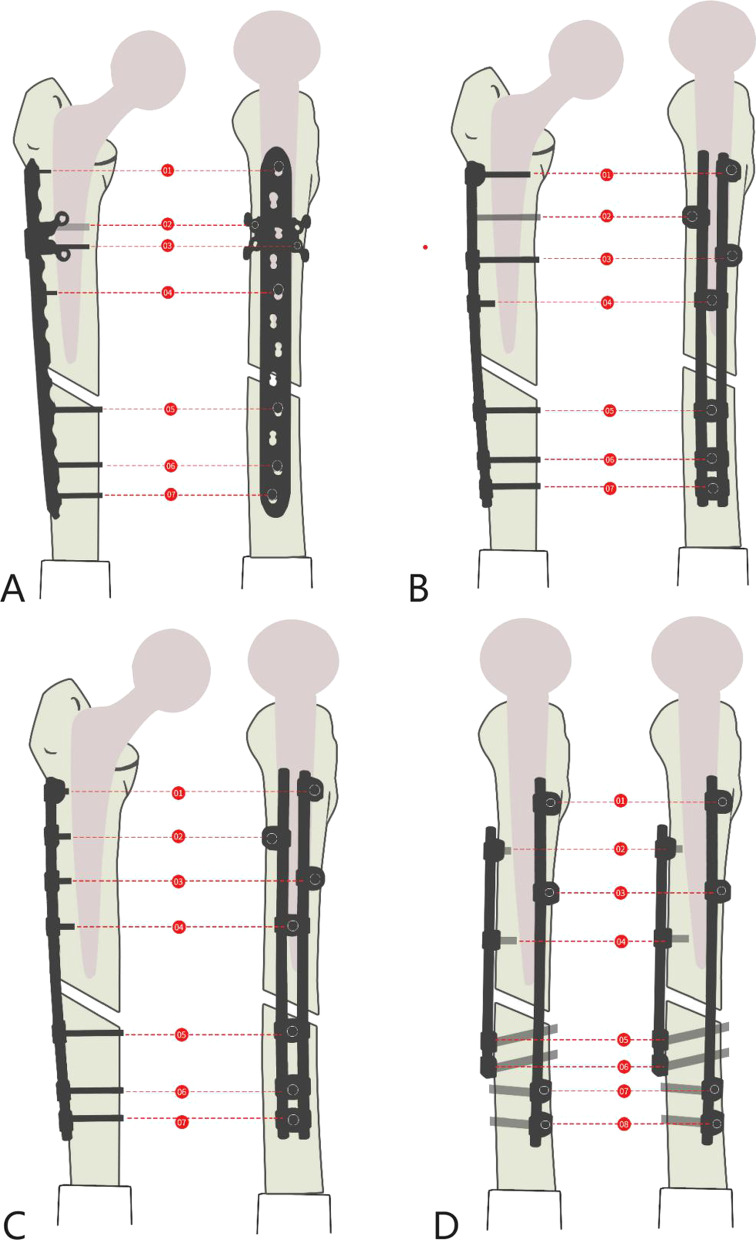
A computer-aided drawing of concrete internal fixation for Groups **A**–**D** of the experimental models

One connecting rod (length, 22 cm; diameter, 6 mm) and one connecting rod with a shorter length were used. Proximal fixation was achieved with four single-rod single-hole fixed blocks and four single-cortex locking screws. Distal fixation was achieved with four double-cortex locking screws. Long connecting rod screws and short connecting rod screws were interlaced at 90° angles (Fig. 3).

### Biomechanical test

Dental tray powder (polymethyl methacrylate) was poured on the distal femur, and an appropriate iron cup was used to fix the distal femur in the experiment. Outside the bone cement and 2.5 cm away from the distal end of the femoral stem prosthesis, an industrial wire saw was then used to cut a 45° oblique fracture line from the upper lateral side to the lower medial side (simulated anatomical reduction and Vancouver B1 simple fracture fixation), so that the supporting effect of internal fixation could be prevented during the axial compression test [24]. All samples were subjected to axial compression and torsion tests, and the stiffness and torsion angles of the different system groups were obtained when treating simple fractures. An industrial milling machine was then used to form a 5 mm bone gap at the sawing bone site to simulate the comminuted fracture model. Two axial compression stiffness groups and the torsion angle data were obtained when testing and treating complex fractures again. Finally, all samples were subjected to axial compression failure, and failure compression force values were obtained. In this experiment, simulated simple fracture refers to the initial osteotomy performed with anatomical reduction, while simulated comminuted fracture refers to the 5 mm gap introduced. In the experiment of axial compression failure, we continuously increased the axial load and observed that the irreversible failure of internal fixation or femur indicated that the experiment was completed [24, 25].

## Experimental tests

### Axial compression test

**Fig. 4:**
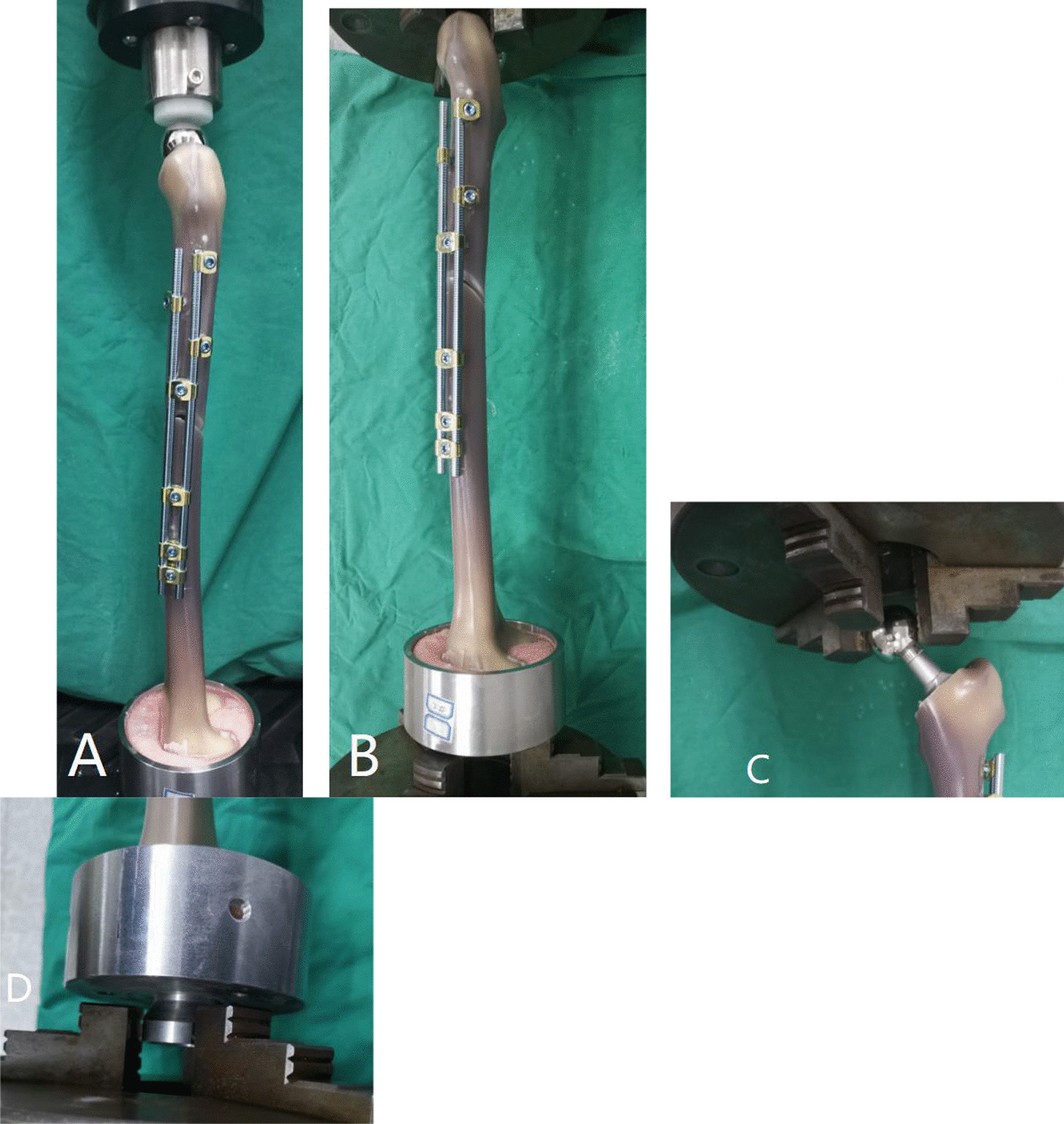
The construction model of OBS on the testing machine. **A** A structural model of the OBS was tested using a microcomputer-controlled electronic universal testing machine, with a cast iron cup at the distal femur matched and fixed to the testing machine, and with the femoral head prosthesis at the proximal femur in contact with the white polyethylene cylinder of the testing machine. **B** The structural model of the OBS was tested on the torsion testing machine. **C** The proximal femoral head was fixed with clamps at both ends of the testing machine. **D** The cast iron cup at the distal femur was matched and fixed to the testing machine

The experiment was performed using a microcomputer-controlled electronic universal testing machine (Equipment model E45.105, Fig. 4). The experiment was performed under an initial vertical load < 100 N, a maximum vertical load of 1000 N, and a displacement loading rate of 8 mm/min, and the load–displacement curve was obtained. The slope of the curve was obtained using computer software (TW-Elite) connected to the testing machine.

### Torsion test

This experiment was performed using a torsion testing machine (Equipment model ND-200; Fig. 6). The experimental conditions comprised a maximum of 10 N^.^m and a loading rate of 90°/min. P-main computer software was connected to the testing machine, which was used to obtain the torque-rotation curve, and a lower load was selected to avoid permanent damage to the samples during testing.

### Axial compression failure test

The axial compression failure test was the final failure test, and it was conducted using a microcomputer-controlled electronic universal testing machine (equipment model: E45.105). The initial vertical load was applied to the femoral head within 100 N, and the displacement loading rate was 8 mm/min until the implant or femur was irreversibly damaged, and the failure mode and verticality upon failure were recorded.

## Experimental evaluation

The stiffness value indicates the capacity of an implant to resist deformation in the elastic stage, and it is used as a standard evaluation of axial compression. Under the same axial pressure, the greater the stiffness value, the smaller the deformation of the plant and the firmer it is. The torsion experiment was used to compare the difference in torsion angle between different implanted structures under the same torque load, that is, torsion stiffness. Under the same torsion force, the torsion angle of the *internal fixation* decreases, which means that the *internal fixation* is stronger. The aim of the axial compression failure test was to detect the maximum load that the structure of the implant could bear. The greater the maximum load, the stronger the resistance of the implant to destructive force and the better the overall strength.

### Statistical analysis

To analyse the differences among all of the structures, after ensuring the normal distribution of the test data, they were analysed using one-way ANOVA, and the significance level was set at *P* < 0.05. When the variance between the control groups maintained homogeneity, the Bonferroni method was used for multiple comparisons in pairs, while the Tamhane method was used for multiple comparisons when the variance between the control groups did not maintain homogeneity. When analysing the correlation between the simulated simple fracture group and the simulated comminuted fracture group, a *t*-test was performed, and the Bonferroni significance level was adjusted to *P* < 0.0125. The adjustment value was calculated by dividing the *P* value of the 95% confidence interval (CI) by the number of constructions compared, that is, Bonferroni = *P* value of the 95% CI/number of constructions = 0.05/4 = 0.0125.

## Results

### Axial stiffness

**Fig. 5:**
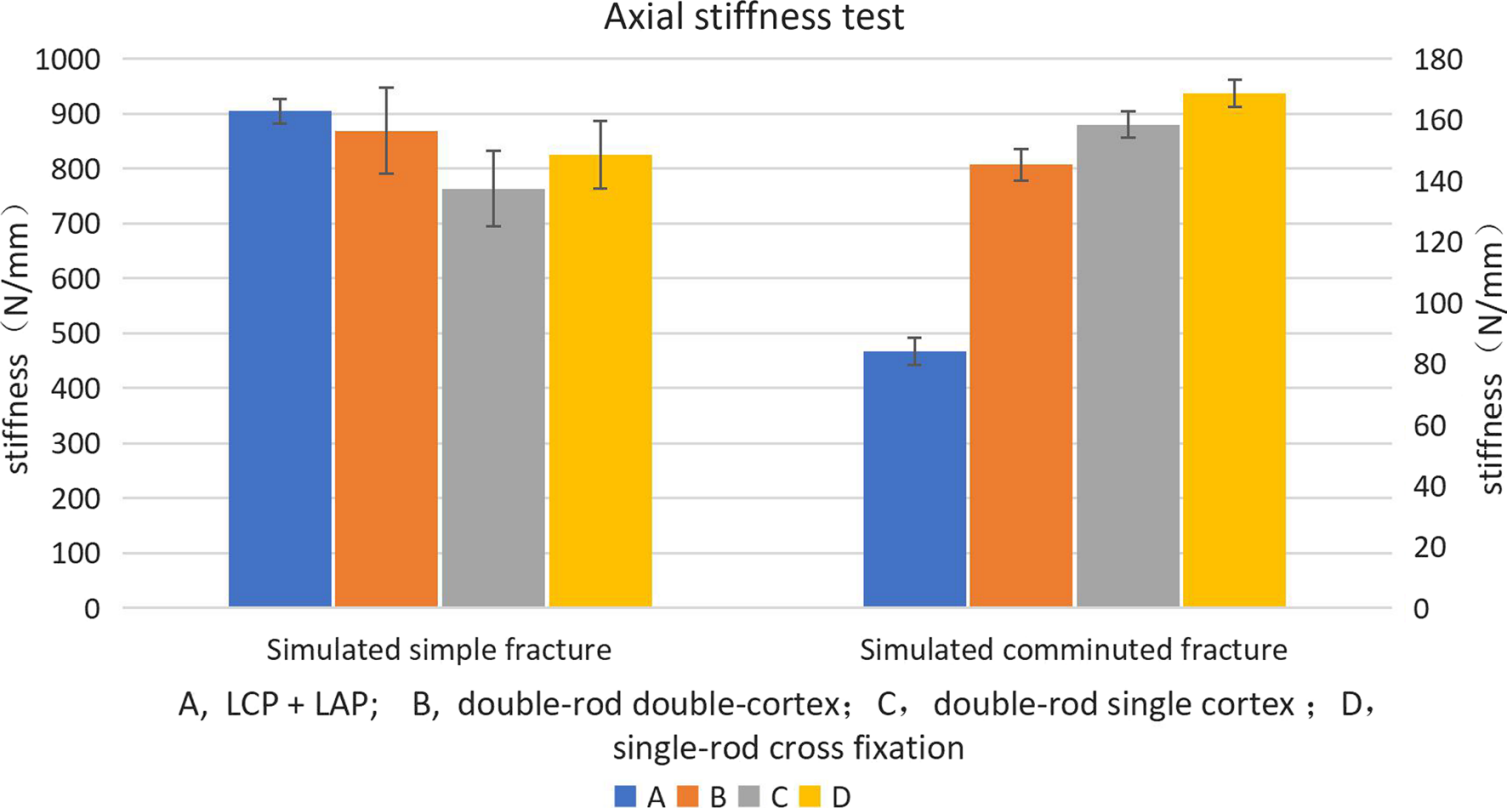
Stiffness results in the axial compression experiment. The error line indicates the standard error of the average value

In the simulation of the simple fracture, the axial stiffness of all structures ranged from 655.9 to 1113.5 N/mm. In the simulation of the comminuted fracture, the axial stiffness ranged from 75.5 to 176.8 N/mm (Fig. 5). In the simulation of the simple fracture, there was no statistically significant difference in the axial stiffness data for each structure (*P* = 0.257). The axial stiffness of the three OBS groups was higher than that of the LCP + LAP group (*P* = 0.000). The axial stiffness of the bridging single-rod cross fixation group was higher than that of the bridging double-rod and double-cortex fixation groups (*P* = 0.02). There were no significant differences in the axial stiffness data of the other groups (*P* > 0.05). When comparing the simulated simple fracture group and the simulated comminuted fracture group, the axial stiffness of each structure in the simple fracture group was higher than the comminuted fracture group (*P* < 0.0125).

### Torsion angle

**Fig. 6:**
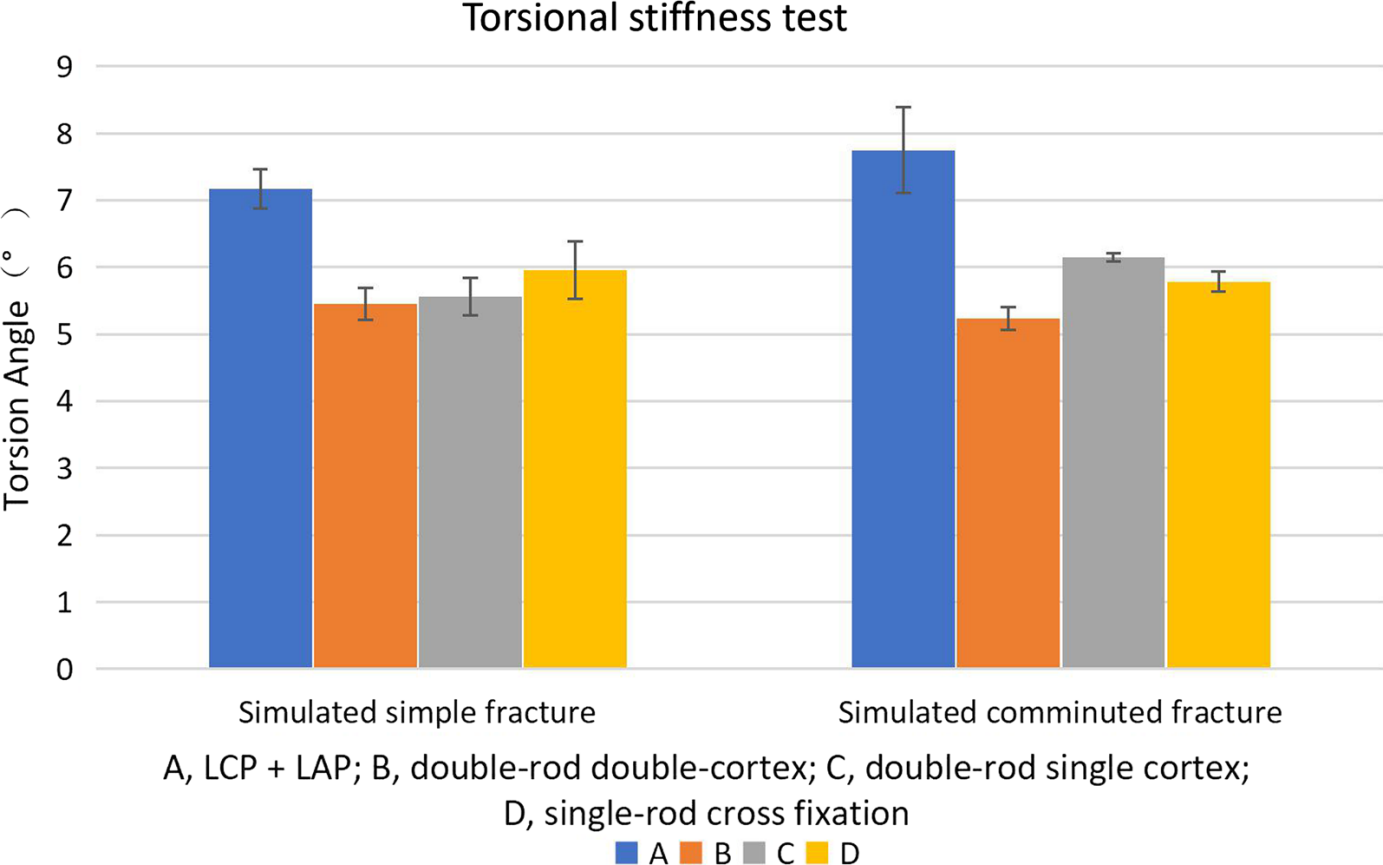
Torsion angle experiment results. The error line indicates the standard error of the average value

In the simulation of the simple fracture, the torsion angles of all structures ranged from 4.53° to 7.88°, and in the simulation of the comminuted fracture, the torsion angles of all structures ranged from 4.8° to 9.18° (Fig. 6). Data analysis during the simulation of simple fractures showed that the torsion angle of the LCP + LAP system was significantly higher than that of the three OBS combinations (*P* < 0.05). Bridge data differences between the three combination types were not statistically significant (*P* > 0.05). In the comminuted fracture simulation data, the LCP + LAP system torsion angle above the bridge double-rod double-cortex (*P* = 0.024) and the bridge double-rod torsion angle of the single-cortex combination were higher than that of the bridging double-rod double-cortex combination (*P* < 0.05). There was no statistically significant difference within the data set, and no statistical significance was found when comparing the simulated simple fracture group and the simulated comminuted fracture group (*P* > 0.0125).

### Axial failure test load

**Fig. 7:**
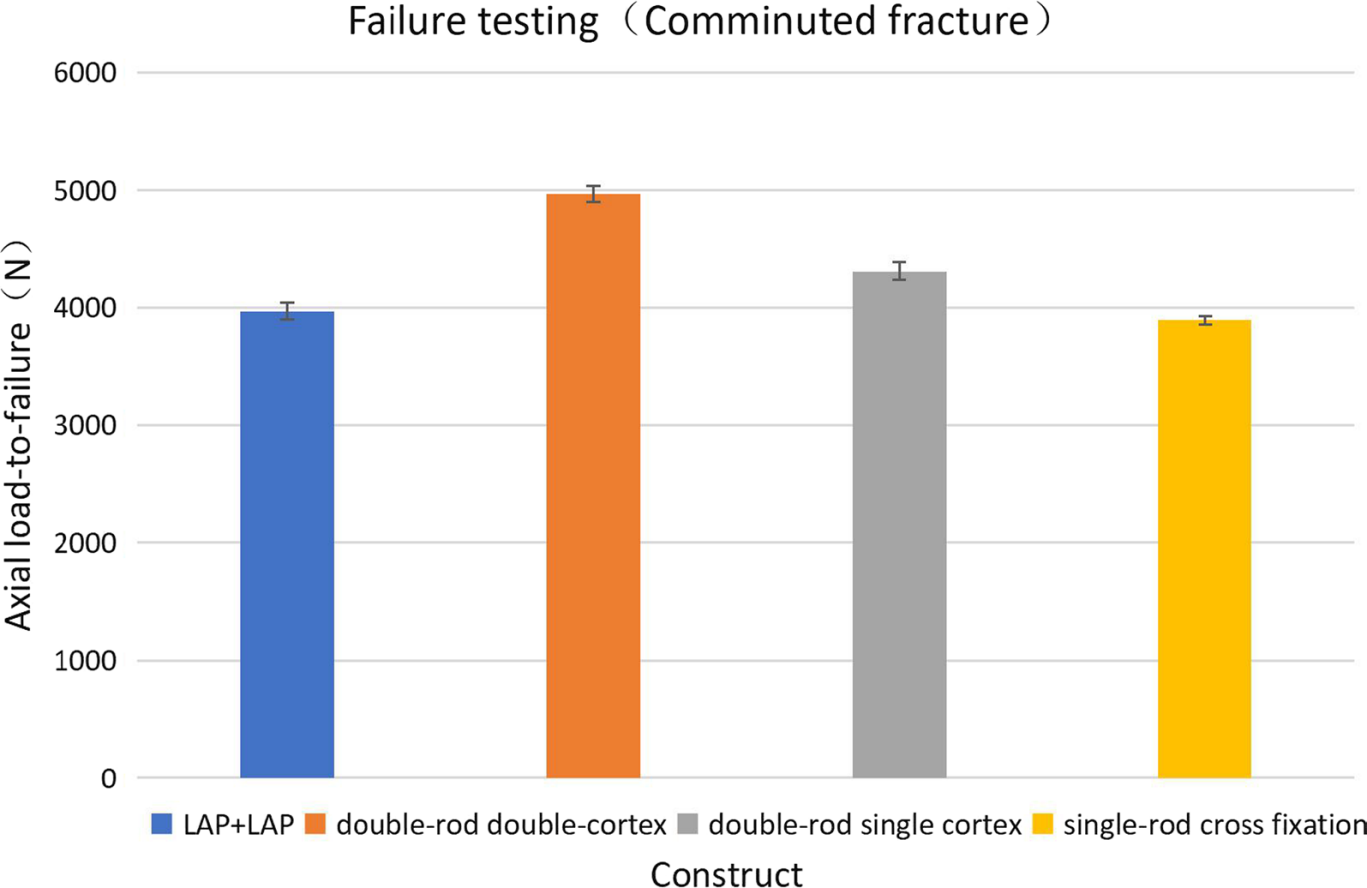
Test pressure values in the axial compression failure experiment. The error line indicates the standard error of the average value

In the final failure test, the axial failure load of the sample ranged from 3756.29  N to 5124.34  N (Fig. 7). The failure pressure of the LCP + LAP system was lower than that of the bridged double-rod double-cortex and bridged double-rod single-cortex combinations (*P* < 0.05). The failure pressure of the bridged double-rod double-cortex combination was higher than that of the bridged double-rod single-cortex and single-rod crossover fixation combination (*P* < 0.05), whereas the failure pressure of the bridged double-rod single-cortex combination was higher than that of the bridged single-rod crossover fixation combination (*P* < 0.05).

In the axial compression failure test, with an increase in the vertical load, the fracture gap decreased as a result of contact between the plate and bridge, which were fixed in a progressive bending position, and irreversible failure subsequently occurred. After all samples were damaged, several failure modes seen in all samples and groups were obtained, although fracture of the permanent plate, attached plate, and connecting rod was not observed. Moreover, no screw was loose, pulled out, or damaged, and the bone cement femoral stem was loose.

**Fig. 8:**
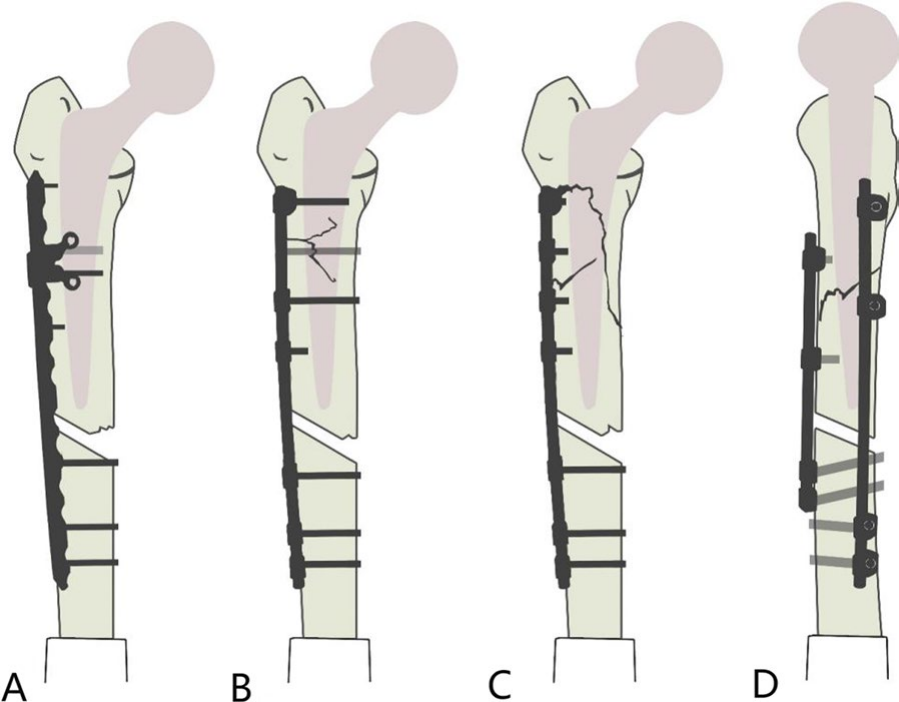
**A** The failure mode of the LCP + LAP group, **B** the failure mode of the bridged two-rod double-cortex group, **C** the failure mode of the bridged two-rod single-cortex group, and **D** the failure mode of the bridged single-rod cross-fixed group

The fracture modes in all groups were similar (Fig. 8), and the failure modes in the LCP + LAP group were similar; that is, there was destruction of the upper cortex at the contact point of the medial femoral space, while no fracture of the femur was found. The failure mode of the OBS in the double-rod and double-cortex group showed not only destruction of the upper cortex at the contact point of the medial bone gap but also a stable transverse crack near the first bicortical screw on the lateral femur and a longitudinal fracture crack between the first bicortical screw and the second screw on the medial femur. This was observed in 3 of 6 samples. The failure mode of the OBS with double rods and a single cortex was similar to that of the combination of double rods and double cortices. Both had cortical failure of the LCP system, which originated from an oblique fracture (from top to bottom) from the first to the third screw in the proximal femur (6 samples) and from a stable transverse crack near the third screw (3 of 6 samples). Cortical damage to the LCP system and the stable oblique crack of the second screw originating from the long rod also occurred in the single-rod cross-fixation group of the OBS.

## Discussion

Treatment of femoral PPFx is a major challenge in modern orthopaedics, and adequate fixation around the prosthesis and the choice of effective internal fixation remains clinically demanding.

This study compared the biomechanics of the LCP + LAP and the OBS for fixation of femoral PPFx. First, a load–displacement curve was obtained through an axial compression test, in which the slope of the stress–displacement curve is considered to demonstrate the stiffness of the internal fixation model as a whole (the fracture end contracts under the load). The initial axial stiffness of the LCP + LAP fixation group was not significantly different from that of the group in Lenz et al.’s study [26]. The stiffness measurements and torsion angles in our study can be used as biomechanical parameters, especially for the OBS. When simulating simple fractures in this study, no apparent difference in stiffness was observed among the four groups in the axial compression experiment (*P* > 0.05); however, the torsion angle of the LCP + LAP fixation group was higher than that of the OBS group (*P* < 0.05), which indicated that the biomechanics of the three OBS combinations were better than those of the LCP + LAP fixation group. When simulating the comminuted fracture, the structural stiffness of the three OBS combination groups was higher than that of the LCP + LAP group (*P* < 0.05), and the torsion angle of the LCP + LAP group was also higher than that of the bridging double-rod and double-cortex group (*P* < 0.05), indicating that the biomechanics of the OBS combination groups were still better than those of the LCP + LAP fixation group. However, when crushing a fracture, the axial compression stiffness of the comminuted fracture group was significantly lower than that of a simple fracture (*P* < 0.0125). Lenz et al. [26] reported that because the support of the cortex at the fracture was reduced, an implant load was needed at this time; therefore, internal fixation was very important for axial stability, especially for crushing a fracture. Finally, the results of the axial compression failure test showed that in OBS, the bridging double-rod and double-cortex fixation and bridging double-rod single cortex fixation have stronger resistance to destructive power than LCP + LAP. However, although the OBS had more fractures on the surface, its failure mode indicated that stress could be dispersed to the femur when the OBS was used in the femur during axial compression. It could be inferred that the failure mode of the locking steel plate was concentrated on the steel plate, which led to the steel plate bearing more stress than the femur. This is consistent with the stress distribution assessment of the finite element analysis of these two systems in the femur by Xiong Ying [17].

Kammerlander et al. [27] showed that patients aged ≥ 75 years who received treatment for hip fractures were unable to maintain postoperative weight-bearing limits, with 69% of patients exceeding more than twice the prescribed partial weight-bearing limits; therefore, the goal of treating periprosthetic fractures must be to facilitate immediate full weight-bearing. During gait, an internal fixation system needs to carry 2 to 2.4 times an individual’s weight [28, 29]. The destructive test results in this study were > 3000 N, which was sustainable. However, the concentration of stress on the plate may cause internal fixation fatigue failure due to high-stress cyclic loading during early functional exercise [30]. The OBS can withstand a higher axial load and distributes axial load to the femur, which could make it more suitable for early functional exercise and fracture healing.

Challenges when treating PPFx after cemented hip arthroplasty concern fixation around the prosthesis. Due to the inconvenience of double-cortex screw placement and the relative imbalance between proximal and distal fixation, pull-out of single cortex screws may occur in internal fixation. Fulkerson et al. [31] observed early failure and axial displacement of the single cortex locking screw under cyclic loading, and other biomechanical studies have shown that double-cortex screw internal fixation has advantages over single cortex screw or cortical ligation alone in proximal fixation of PPFx [26, 32–36]. In this study, double-cortex fixation of the OBS was guided using a C-arm machine. Moreover, because of the orientation and the corresponding angle of the OBS, the connecting block could be adjusted to achieve flexible screw placement, and double-cortex fixation of the OBS could be undertaken around the prosthesis without using additional devices. In the OBS bridging single-cortex fixation group, proximal fractures were fixed with single-cortex screws, but no proximal screw pull-out was observed during the fracture test, and the biomechanics were similar to that of the LCP + LAP fixation group. Using an OBS flexible connecting rod in the design for the bridge single-rod cross-fixation group, two fixed-rod screws can be placed at 90° in spatial distribution to achieve better stability of the fixed-rod screw. However, proximal fractures still occurred with single cortex fixation, although with no screw pull-out damage during the experiment. In the axial compression experiments, there was no significant difference in stiffness between the LCP + LAP fixation group and the bridge single-rod cross-fixation group; however, the anti-torsion performance was better. Insertion of the double-cortex screw was more resistant to pull out under cyclic loading, which is conducive to functional exercise in early rehabilitation and avoids screw pull-out failure [33].

It has been reported that minimally invasive percutaneous plate osteosynthesis combined with LCP can be used as a surgical method for the treatment of Vancouver type B1 PPFx, with intraoperative advantages (shorter operation time and less blood loss) [37], and that the OBS can also be combined with Mippo technology for fracture treatment. Liangqi et al. [19] reported that the OBS combined with Mippo technology to treat ipsilateral proximal femoral and diaphyseal fractures had a high possibility of healing fractures and having a good functional effect. The OBS can also provide personalised internal fixation when combined with 3D printing applications for complex and challenging fractures around the prosthesis, with preoperative preparation having obvious advantages. Moreover, the OBS has many choices of fixed combinations, allowing greater flexibility for the operating surgeon, who can then obtain the best implant position, especially around the prosthetic implants.

This study had several limitations. A standard artificial femur was used in this study, which could simulate good bone reserve. Models for osteoporosis are available; however, this study did not simulate osteoporosis and low-quality bone. Patients with fractures around a femoral prosthesis comprised older adults, and these patients commonly have osteoporosis. Furthermore, in vitro simulation experiments, the influence of soft tissue involvement, the short- and long-term role of soft tissue in fracture stabilization and healing, and the convenience of OBS placement and screw placement could not be evaluated. The effects of cyclic loading were also not tested in this study.

## Conclusions

This study showed that the axial stiffness of the three OBS combinations did not significantly differ from that of the LCP + LAP system in simple fractures around the femoral prosthesis; however, torsion resistance was higher in the three OBS combinations than in the LCP + LAP system. In the simulation of comminuted fracture, the axial stiffness of the OBS was better than that of the plate system, and the torsion resistance of the bridging double-rod and double-cortex system was outstanding. The failure mode of the failed experiment showed that the stress of the OBS was more dispersed, which was consistent with the findings of previous studies. The overall biomechanics of the OBS combinations was good, and the diversity and flexibility of the OBS combinations provided a range of treatment options. The next step will be to continue clinical research to evaluate its effectiveness and practical value.

## Data Availability

The data that support the findings of this study are available from the corresponding author upon reasonable request.

## Abbreviations

LCP + LAP: Locking compression plate/locking attachment plate structure
OBS: Ortho-Bridge System
ORIF: Open reduction and internal fixation
PPFx: Periprosthetic fracture
